# ADVANCE DIRECTIVES: STATE REQUIREMENTS, PRACTICES, AND PREVALENCE IN ADULT DAY SERVICES CENTERS

**DOI:** 10.1093/geroni/igz038.566

**Published:** 2019-11-08

**Authors:** Jessica P Lendon, Christine Caffrey, Denys Lau

**Affiliations:** National Center for Health Statistics, Hyattsville, Maryland, United States

## Abstract

Advance directives (ADs) are documents that express a person’s healthcare preferences if he/she is unable to make decisions. Adult day service centers (ADSC) may serve as an entrée to advance care planning for many people. This study examined the relationships among: 1-state requirements on ADSCs to provide information on ADs; 2-ADSC’s awareness of their state requirement; 3-ADSC’s practice in providing AD information; and 4-the percentage of ADSC participants with an AD. From the 2016 National Study of Long-Term Care Providers, 3,300 ADSCs reported that they maintained documentation of ADs in participants’ files. Nine states required ADSCs to provide information on ADs; 22% of ADSCs were located in these states. About 24% of ADSCs did not know if their states had requirements; among the 76% of ADSCs that reported knowing, 62% were correct and 37% were incorrect. About 80% of ADSCs provided AD information to their participants, while 41% of ADSC participants had an AD on file. Regression models controlled for size, chain and profit statuses, Medicaid-licensing, medical or social care model, electronic health records use, and Census region. Having state requirements was not independently associated with ADSCs’ practice of providing AD information or with the percentage of participants with an AD. Instead, ADSCs that thought their state had a requirement had greater odds of providing information on ADs, regardless of state requirements. Similarly, ADSCs that thought their state had a requirement and that provided AD information had a higher percentage of participants with an AD, independent of state requirements.

